# Neutrophils in chronic lymphocytic leukemia are permanently activated and have functional defects

**DOI:** 10.18632/oncotarget.20031

**Published:** 2017-08-08

**Authors:** Gayane Manukyan, Tomas Papajik, Petr Gajdos, Zuzana Mikulkova, Renata Urbanova, Gabriela Gabcova, Milos Kudelka, Peter Turcsányi, Pavlina Ryznerova, Vit Prochazka, Eva Kriegova

**Affiliations:** ^1^ Department of Immunology, Faculty of Medicine and Dentistry, Palacky University Olomouc, Olomouc, Czech Republic; ^2^ Department of Hemato-Oncology, Faculty of Medicine and Dentistry, Palacky University and University Hospital, Olomouc, Czech Republic; ^3^ Department of Computer Science, Faculty of Electrical Engineering and Computer Science, VSB-Technical University of Ostrava, Ostrava, Czech Republic; ^4^ Laboratory of Molecular and Cellular Immunology, Institute of Molecular Biology NAS RA, Yerevan, Armenia

**Keywords:** chronic lymphocytic leukemia, neutrophils, disease activity, flow cytometry, enhanced ROS production

## Abstract

A growing body of studies highlights involvement of neutrophils in cancer development and progression. Our aim was to assess the phenotypic and functional properties of circulating neutrophils from patients with chronic lymphocytic leukemia (CLL). The percentage of CD54+ and CD64+ neutrophils as well as CD54 expression on these cells were higher in CLL patients than in age-matched healthy controls. Neutrophils from CLL produced more reactive oxygen species (ROS) compared to controls in both resting and activated conditions. Lipopolysaccharide-induced production of IL-1β and TNF-a as well as reduced TLR2 expression in neutrophils from CLL than in neutrophils from controls suggesting their tolerant state. Finally, phenotypic alterations of neutrophils, particularly elevation of CD64 and CD54 markers, correlated with disease activity and treatment, and low percentage of neutrophils. Taken together, the alterations in percentage and functional characteristics of neutrophils reflect the clinical course of CLL. Our data provide first evidence that neutrophils in CLL are permanently primed and have functional defects.

## INTRODUCTION

Chronic lymphocytic leukemia (CLL) is characterized by an accumulation of abnormal B lymphocytes in the bone marrow, lymphoid tissues and peripheral blood. The expansion of malignant clone(s) may in turn lead to phenotypic alterations of residual hematopoietic bone marrow cells such as those maturing neutrophils, monocytic and/or erythroid cells as well as CD34+ myeloid precursors; and this may be also reflected in circulating cells as shown already in myeloid malignancies [1, 2]. Whether such phenotypic alterations are also present in circulating neutrophils in CLL patients remains to be investigated.

Polymorphonuclear neutrophils are the most abundant phagocytes in the circulation and are traditionally associated with innate defense against infection [3, 4]. When activated, they release a spectrum of inflammatory mediators, produce reactive oxygen species (ROS), activate complement and regulate inflammation [5]. Besides, neutrophils mediate antibody-dependent cell-mediated cytotoxicity (ADCC) [6, 7]. A growing body of studies shows that their inappropriate activation is associated with inflammatory, autoimmune processes as well as cancer development and progression [8].

The circulating neutrophils from CLL patients were shown to possess impaired bactericidal activity [9], probably due to myeloperoxidase deficiency and impaired migratory abilities [10]. More recently, neutrophils were shown to differentiate toward a B-cell helper phenotype in murine model of CLL contributing to the formation of survival niches for CLL cells in lymph nodes [11]. Nevertheless, neutrophils in CLL may contribute to treatment response as shown by phagocytosis of about 50% of anti-CD20 (obinutuzumab, but not rituximab) opsonized CLL targets by drug-activated neutrophils [12].

In an attempt to further understand how neutrophils may contribute to CLL pathogenesis, we aimed to assess the phenotypic and functional properties of circulating neutrophils from patients with CLL. We investigated expression profiles of major membrane-bound markers associated with cell activation: i) surface adhesion molecules associated with inflammation (CD11b, CD62L, CD54), and ii) Fcγ receptor: FcγRI (CD64) using flow cytometry. Further, we determined the priming ability as well as baseline and stimulated generation of ROS by the neutrophils obtained from CLL patients.

## RESULTS

### Surface molecule expression on neutrophils

In order to characterize the circulating neutrophils in CLL, we investigated their immunophenotypes using following surface molecules CD54, CD11b, CD62L, and CD64 and compared with those on neutrophils from age-matched healthy subjects. Neutrophils were selected in the forward *versus* side scatter dot plot, and additionally gated as CD15/CD16 positive cells (Supplementary Figure 1 in the on-line Supplement).

**Figure 1:**
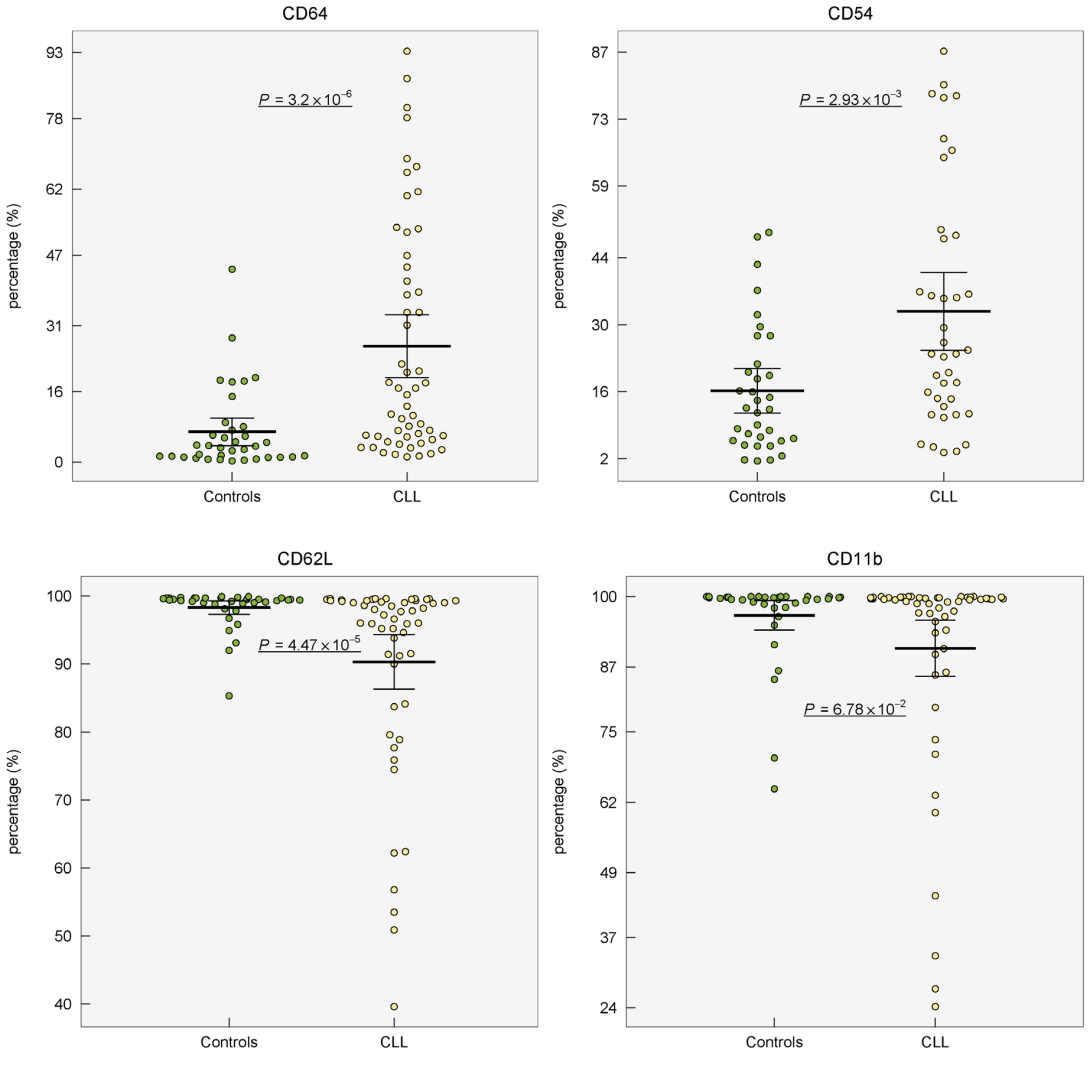
Distribution of percentage of surface markers (CD64, CD54, CD62L, CD11b) on circulating neutrophils from healthy controls and patients with CLL Group means are indicated by horizontal bars, error bars indicate 95%CI; *P* values for differences between two groups are stated.

Expression of marker CD54 was increased (*P* < 0.001) and higher percentage (*P* < 0.01) of CD54+ neutrophils was observed in CLL patients compared to healthy controls (Table 1, Figure 1 Supplementary Figure 2 in the on-line Supplement). When controls and CLL were compared, the percentage of CD64+ cells was 4-fold higher (*P* < 0.001) in CLL patients showing marked inter-individual variability ranging from 1.2 to 93.3%. The expression (MFI) of CD64 did not differ between CLL *vs* controls (*P* = 0.71). Lower percentage of CD62L+ cells (*P* < 0.001) as well as lower expression of CD62L (*P* = 0.02) was found in neutrophils from CLL patients comparing to age-matched healthy controls (Table 1, Figure 1, Supplementary Figure 2 in on-line Supplement). Expression of CD11b on neutrophils did not vary between CLL patients and controls (*P* > 0.05). The comparison of expression of studied surface markers on neutrophils from healthy subjects and CLL patient subgroups according to the treatment history is shown in Supplementary Table 1 in on-line Supplement.

**Table 1:** Relative and absolute neutrophil counts and expression levels of surface markers on neutrophils in: A. healthy controls *vs* CLL, B. non-active CLL *vs* active CLL, C. untreated CLL *vs* treated CLL, D. mutated *IGHV* gene status *vs* unmutated *IGHV* gene status.

*Marker*	*Mean (95% CI)*		*FC#*	*P*
**A**	***Healthy controls***	***CLL***		
CD64 (%)	6.89 (3.73-10.0)	26.3 (19.2-33.5)	4.65	3.20 × 10^-6^
CD54 (%)*	16.2 (11.5-20.9)	32.8 (24.6-41.0)	1.92	2.93 × 10^-3^
CD62L (%)	98.3 (97.3-99.3)	90.3 (86.3-94.3)	0.98	4.47 × 10^-5^
CD11b (%)	96.5 (93.8-99.3)	90.5 (85.3-95.7)	0.99	0.07
CD64 (MFI)	58.8 (51.4-66.3)	60.3 (51.8-68.8)	0.89	0.71
CD54 (MFI)*	16.4 (15.9-17.0)	20.1 (18.0-22.1)	1.19	4.92 × 10^-7^
CD62L (MFI)	423 (334-512)	302 (246-358)	0.87	0.03
CD11b (MFI)	120 (103-138)	148 (125-171)	1.28	0.14
Neutrophils (%)	58.4 (56.2-60.7)	16.9 (13.0-20.8)	0.23	4.04 × 10^−15^
Lymphocytes (%)	28.8 (26.9-30.8)	75.2 (70.7-79.6)	2.73	3.91 × 10^−15^
ANC (x10^9^/L)	4.82 (4.09-5.55)	4.19 (3.62-4.75)	0.76	0.10
CD64+ ANC (x10^9^/L)	0.40 (0.08-0.71)	1.06 (0.68-1.44)	3.12	4.27 × 10^−3^
CD54+ ANC (x10^9^/L)	0.59 (0.33-0.85)	1.25 (0.90-1.60)	2.56	4.14 × 10^−3^
CD62L+ ANC (x10^9^/L)	4.79 (4.07-5.51)	3.82 (3.29-4.35)	0.73	0.03
CD11b+ ANC (x10^9^/L)	4.81 (4.08-5.54)	3.87 (3.28-4.47)	0.74	0.04
**B**	***CLL non-active***	***CLL active***		
CD64 (%)	15.7 (8.68-22.8)	40.0 (27.8-52.1)	4.91	3.01 × 10^-3^
CD54 (%)*	27.9 (17.7-38.2)	38.5 (24.8-52.2)	1.54	0.21
CD62L (%)	93.1 (88.2-98.1)	88.3 (81.9-94.7)	0.94	8.11 × 10^-3^
CD11b (%)	91.4 (84.2-98.6)	90.5 (82.4-98.6)	0.99	0.86
CD64 (MFI)	50.5 (43.4-57.6)	69.9 (53.7-86.1)	1.26	6.09 × 10^-3^
CD54 (MFI)*	17.9 (17.0-18.9)	22.6 (18.3-26.8)	1.06	2.93 × 10^−3^
CD62L (MFI)	363 (281-445)	237 (165-310)	0.51	0.03
CD11b (MFI)	145 (111-179)	146 (114-178)	1.02	0.70
Neutrophils (%)	23.4 (17.7-29.1)	8.83 (5.07-12.6)	0.32	6.26 × 10^−5^
Lymphocytes (%)	68.9 (62.4-75.5)	82.7 (77.6-87.8)	1.20	1.38 × 10^−3^
CLL cells (%)	60.3 (49.0-71.6)	80.5 (72.0-89.0)	1.23	2.67 × 10^−3^
ANC (x10^9^/L)	4.42 (3.69-5.14)	3.94 (2.97-4.92)	0.82	0.25
CD64+ ANC (x10^9^/L)	0.65 (0.33-0.97)	1.59 (0.86-2.33)	3.45	9.52 × 10^−3^
CD54+ ANC (x10^9^/L)	1.17 (0.65-1.69)	1.34 (0.83-1.85)	1.33	0.36
CD62L+ ANC (x10^9^/L)	4.07 (3.39-4.74)	3.60 (2.70-4.49)	0.87	0.24
CD11b+ ANC (x10^9^/L)	4.12 (3.33-4.91)	3.65 (2.68-4.63)	0.78	0.38
**C**	***CLL untreated***	***CLL treated***		
CD64 (%)	16.5 (9.12-23.9)	35.5 (24.1-46.9)	3.52	0.02
CD54 (%)*	23.3 (14.0-32.5)	42.8 (29.9-55.8)	2.07	0.01
CD62L (%)	93.0 (88.3-97.6)	87.8 (81.2-94.4)	0.98	0.18
CD11b (%)	94.6 (90.3-98.9)	86.6 (77.3-95.9)	0.99	0.17
CD64 (MFI)	45.6 (40.5-50.8)	73.8 (59.5-88.1)	1.58	3.51 × 10^−5^
CD54 (MFI)*	18.6 (17.6-19.5)	21.7 (17.4-25.9)	1.03	0.22
CD62L (MFI)	338 (243-433)	269 (203-336)	0.91	0.40
CD11b (MFI)	156 (118-193)	140 (111-170)	0.82	0.37
Neutrophils (%)	18.0 (12.7-23.3)	15.9 (9.81-22.0)	0.81	0.29
Lymphocytes (%)	76.6 (71.0-82.2)	73.8 (66.5-81.0)	0.97	0.80
CLL cells (%)	69.0 (59.5-78.6)	69.5 (57.4-81.6)	1.13	0.53
ANC (x10^9^/L)	5.01 (4.14-5.87)	3.42 (2.76-4.09)	0.71	3.95 × 10^−3^
CD64+ ANC (x10^9^/L)	0.94 (0.29-1.59)	1.18 (0.73-1.63)	2.35	0.15
CD54+ ANC (x10^9^/L)	1.09 (0.54-1.64)	1.41 (0.94-1.89)	1.82	7.90 × 10^−2^
CD62L+ ANC (x10^9^/L)	4.56 (3.82-5.31)	3.13 (2.44-3.82)	0.65	2.51 × 10^−3^
CD11b+ ANC (x10^9^/L)	4.76 (3.90-5.62)	3.05 (2.31-3.78)	0.64	5.72 × 10^−3^
**D**	***CLL IGHV*** ***mutated status***	***CLL IGHV unmutated status***		
CD64 (%)	17.2 (9.30-25.0)	32.0 (19.8-44.1)	1.74	0.23
CD54 (%)*	28.8 (17.5-40.1)	38.3 (23.5-53.0)	1.03	0.37
CD62L (%)	93.5 (87.5-99.5)	87.4 (81.1-93.7)	0.96	9.89 × 10^-4^
CD11b (%)	93.2 (86.0-100)	88.8 (81.1-96.5)	0.98	0.16
CD64 (MFI)	55.0 (43.8-66.1)	66.2 (51.1-81.4)	1.16	0.12
CD54 (MFI)*	18.5 (17.5-19.4)	21.7 (16.9-26.5)	1.03	0.27
CD62L (MFI)	389 (283-494)	235 (173-296)	0.67	0.02
CD11b (MFI)	148 (121-175)	152 (109-195)	0.79	0.62
Neutrophils (%)	21.1(14.7-27.4)	13.5 (8.34-18.7)	0.58	0.04
Lymphocytes (%)	72.8 (64.9-80.7)	76.5 (70.7-82.4)	1.09	0.52
CLL cells (%)	65.0 (52.8-77.3)	71.3 (59.8-82.88)	1.17	0.25
ANC (x10^9^/L)	3.95 (3.29-4.61)	4.42 (3.42-5.41)	1.07	0.76
CD64+ ANC (x10^9^/L)	0.65 (0.27-1.04)	1.44 (0.74-2.15)	2.65	0.12
CD54+ ANC (x10^9^/L)	1.12 (0.58-1.67)	1.47 (0.84-2.09)	1.56	0.18
CD62L+ ANC (x10^9^/L)	3.72 (3.02-4.41)	3.91 (3.02-4.76)	0.96	0.91
CD11b+ ANC (x10^9^/L)	3.72 (3.00-4.44)	4.01 (2.99-5.03)	1.04	0.78

ANC: Absolute neutrophil count. The normal range for the ANC = 1.5 to 8.0x10^9^/L.

#FC (Fold Change) between group medians

*CD54 available in 39 patients and 34 controls

### Baseline expression of cell surface molecules on neutrophils in CLL subgroups

**Figure 2:**
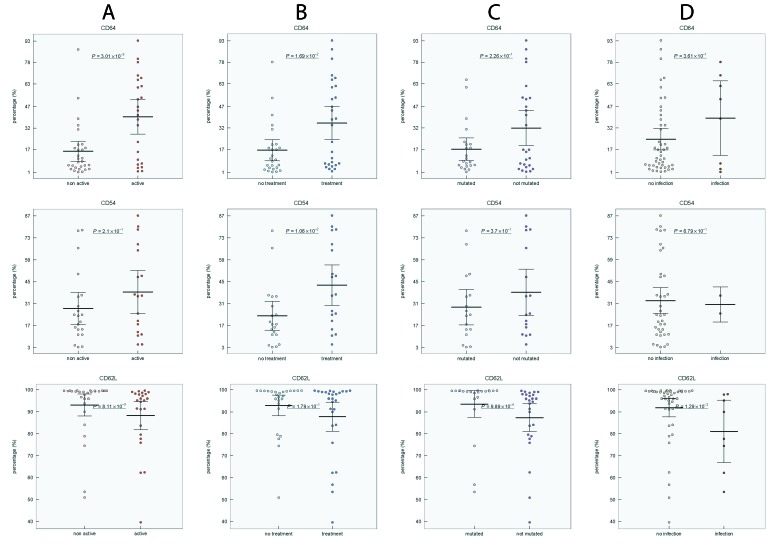
Distribution of CD64, CD54 and CD62L positive neutrophils in CLL subgroups **A.** non-active *vs* active disease, **B.** untreated *vs* treated disease, **C.** mutated *vs* unmutated *IGHV* gene status, **D.** CLL patients without infection *vs* with ongoing infection. Group means are indicated by horizontal bars, error bars indicate 95%CI; *P* values for differences between two groups are stated.

**Figure 3:**
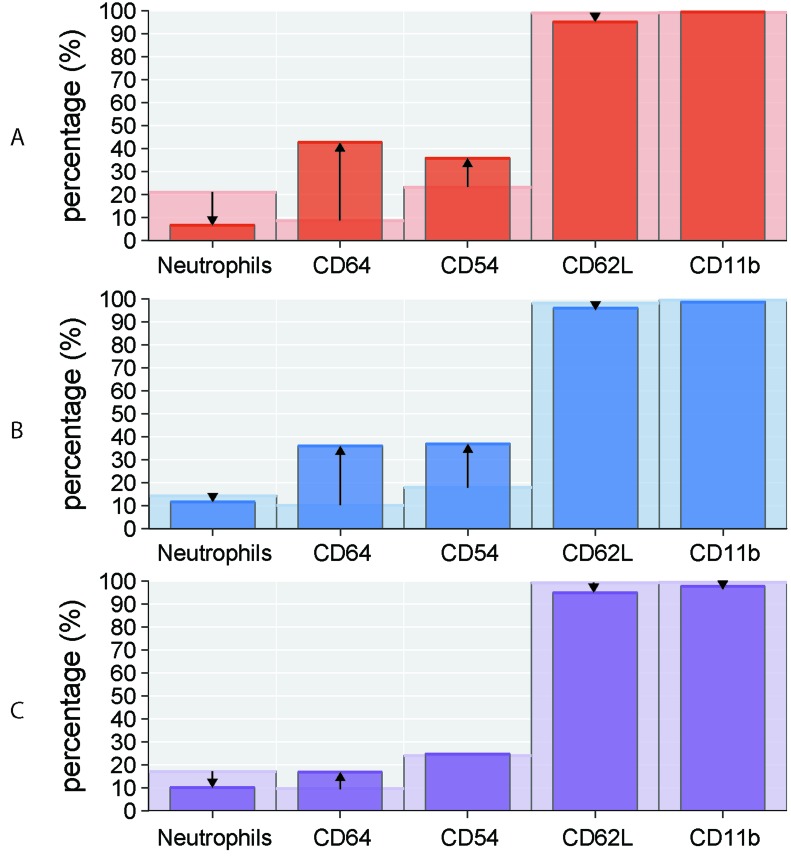
Percentage of circulating neutrophils and surface markers (CD64, CD54, CD62L, CD11b) in CLL subgroups Comparison of studied parameters (medians) between **A.** non-active (light red columns) *vs* active (dark red columns) disease, **B.** untreated (light blue columns) *vs* treated (dark blue columns) disease, **C.** mutated (light violet columns) *vs* unmutated (dark violet columns) *IGHV* gene status. The arrows indicate the increase/decrease in studied parameters in non-active/untreated/mutated CLL subgroups compared to active/treated/unmutated subgroups, respectively.

To determine whether disease activity, treatment, *IGHV* mutational status, Binet stage, blood number counts, and percentage of CLL cells affect the expression level of investigated markers on neutrophils, we compared the expression of CD54, CD11b, CD62L, and CD64 in CLL subgroups. Active disease was associated with upregulated expression of CD54 and CD64 (*P* < 0.01 and *P* < 0.01, respectively), increased percentage of CD64 (*P* < 0.01) and downregulated expression (*P* < 0.05) and percentage (*P* < 0.01) of CD62L (Figure 2A, Figure 3A, Supplementary Figure 3 in on-line Supplement). Treated CLL patients displayed simultaneously increased expression of CD64 (*P* < 0.001), higher percentage of CD64 (*P* < 0.05) and CD54 (*P* < 0.01) compared to untreated patients. Remarkable differences were observed for the percentage of CD64 cells, which were almost 4-fold higher in treated *vs* untreated patients (Figure 2B, Figure 3B, Supplementary Figure 3 in on-line Supplement). When patient subgroups were compared according to Binet stage, the percentage of CD64+ cells was lower in patients with Binet stage A comparing to stages B and C (*P* < 0.05) (data not shown). In patients with ongoing infection, lower percentage of CD11b+, CD62L+ cells and expression of CD62L were detected comparing to those with no infection (Figure 2D, Supplementary Figure 3 in on-line supplement). Patients with unmutated *IGHV* gene status had decreased percentage of CD62L (*P* < 0.001) and a decreased MFI of CD62L (*P* < 0.05) compared with mutated *IGHV* status (Figure 2C, Supplementary Figure 3 in on-line Supplement).

In our study, the percentage of circulating neutrophils in CLL showed a negative correlation with CD64 percentage and MFI (*r* = 0.45, *P* < 0.01 and *r* = 0.24, *P* = 0.07, respectively) and CD54 percentage and MFI (*r* = 0.34, *P* < 0.05 and *r* = 0.43, *P* < 0.01, respectively). In opposite, neutrophil percentage positively correlated with CD62L (%) and MFI (*r* = 0.41, *P* < 0.01 and *r* = 0.46, *P* < 0.01, respectively) (Supplementary Figure 4 in on-line Supplement).

### Cellular pattern of peripheral blood in CLL patients

Further, we compared percentage of circulating neutrophils, lymphocytes and CLL cells (as assessed by CD5+/CD19+) in patient subgroups according to the disease activity, treatment history and *IGHV* gene mutational status. Majority of enrolled CLL patients had decreased percentage of circulating neutrophils compared to healthy subjects (Table 1). The absolute number of neutrophils in CLL patients did not differ from those in healthy controls (Table 1), only 3 patients had absolute neutropenia ( < 2.0 x 10^9^/L). Active disease was associated with a increased of neutrophil percentage (*P* < 0.001) and increased percentage of lymphocytes (*P* < 0.01) and CLL cells (*P* < 0.01) comparing to non-active disease. Patients with unmutated *IGHV* gene status had lower percentage of neutrophils than those with mutated *IGHV* (*P* < 0.05), the difference in neutrophils between treated and untreated patients did not reach significance (Table 1, Supplementary Figure 5 in the on-line Supplement).

### Characteristics of neutrophils associated with CLL and its subgroups

To better characterize the neutrophils in CLL, we determined which markers discriminate i) CLL and healthy controls and ii) CLL subgroups according to the disease activity, treatment history and *IGHV* gene mutational status. We calculated combinations of every two and three markers (percentage and MFI of studied surface markers, percentages of neutrophils, lymphocytes and CLL cells) from individual patients and determined the misclassification error for a particular subgroup.

**Figure 4:**
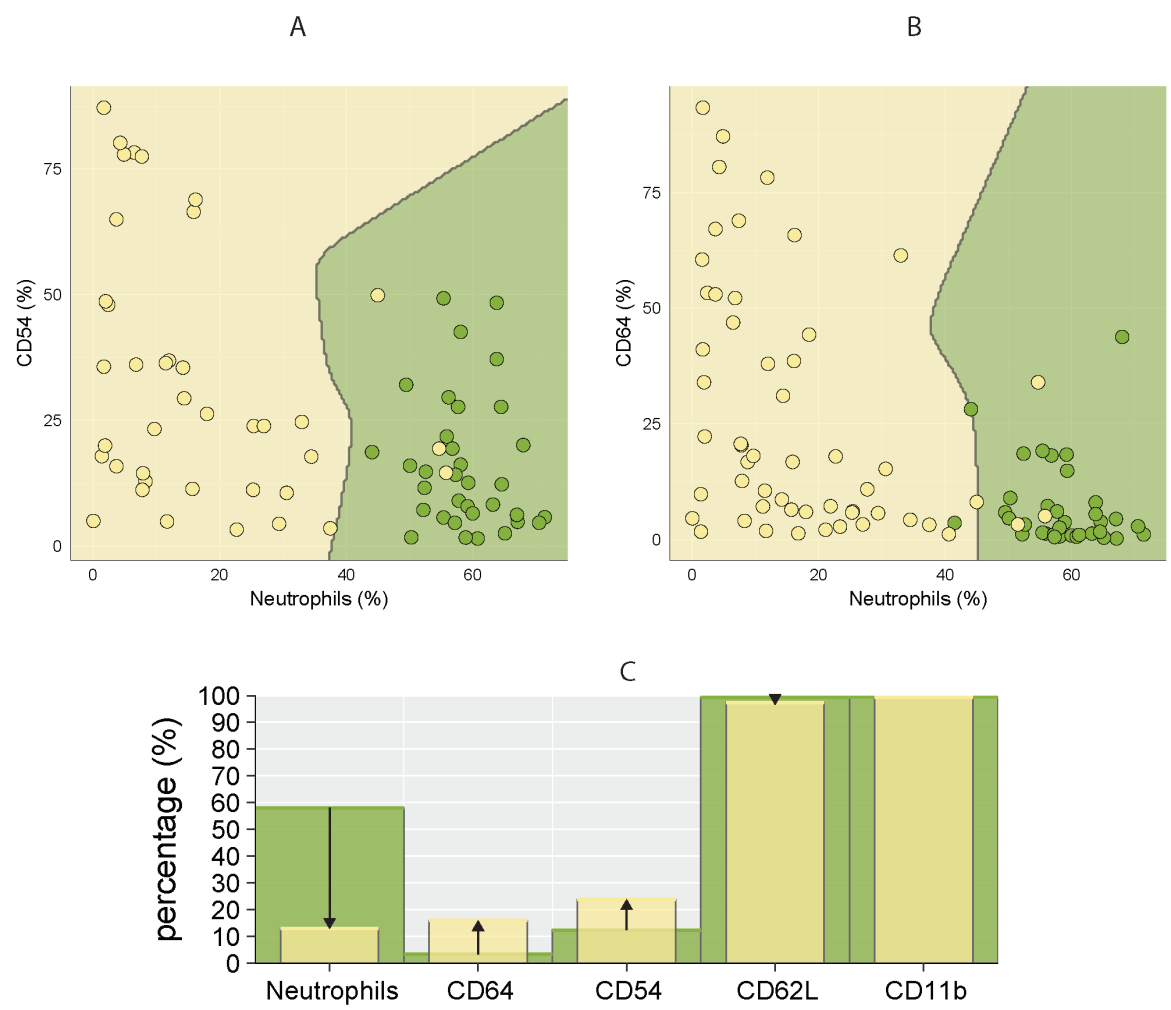
Comparison of percentage of circulating neutrophils and surface markers (CD64, CD54, CD62L, CD11b) in CLL patients and healthy controls CLL is coloured yellow, control subjects green. Discrimination between CLL and controls using combination of two parameters: **A.** CD54 (%) - neutrophils (%) and **B.** CD64 (%) - neutrophils (%). The dots represent the percentage of CD54/CD64 (y-axis) and neutrophils (x-axis) in individual subjects. **C.** Comparison of studied parameters (medians) between CLL (yellow columns) and healthy groups (green columns). The arrows indicate the increase/decrease in studied parameters in CLL patients compared to healthy controls.

**Figure 5:**
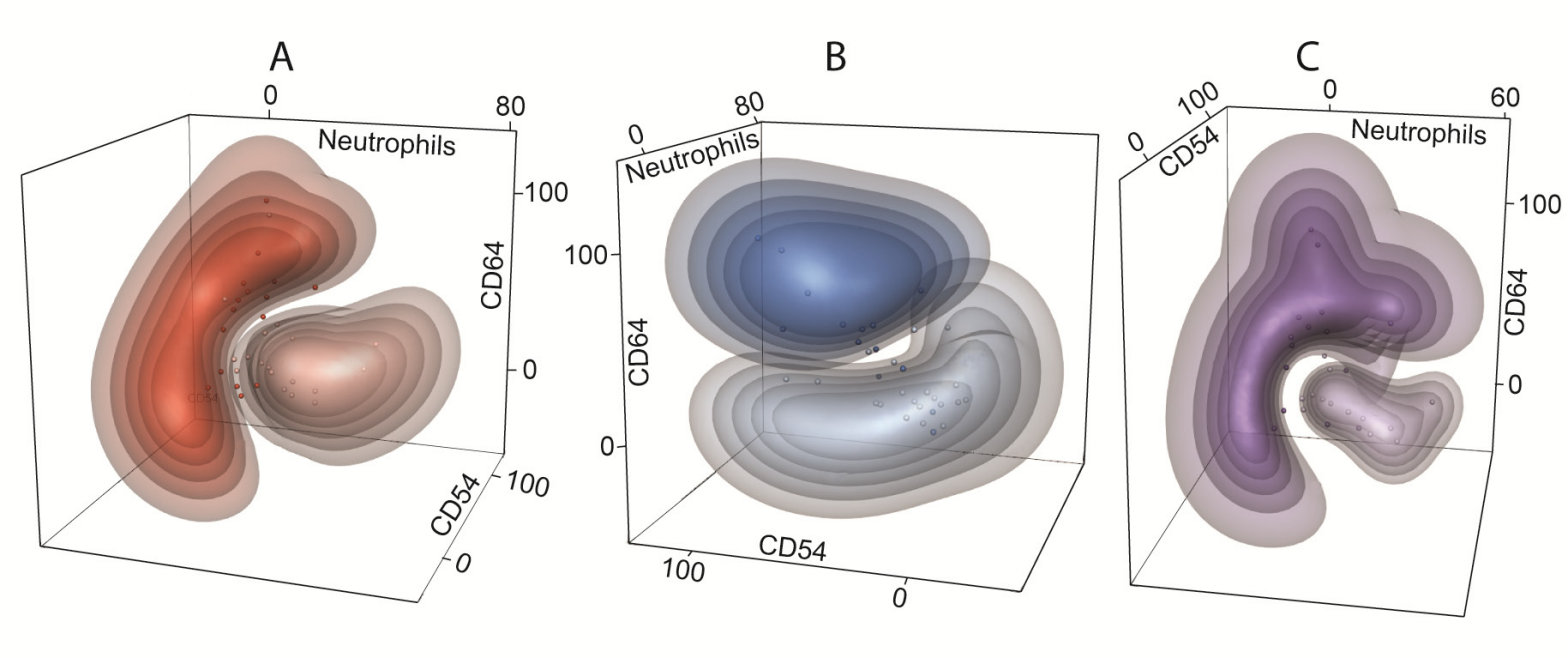
Multidimensional clustering of CLL subgroups Discriminant 3-D models with combination of CD64 (%) - CD54 (%) - neutrophils (%) show the separation of following CLL subgroups: **A.** non-active (light red clouds) *vs* active (dark red clouds) disease, **B.** untreated (light blue clouds) *vs* treated (dark blue clouds) disease, **C.** mutated (light violet clouds) *vs* unmutated (dark violet clouds) *IGHV* gene status. The dots represent the percentage of CD54 (x-axis), CD64 (y-axis) and neutrophils (z-axis) of individual subjects. The colour bulks represent the probability intervals: the more saturated color the higher probability (intervals: > 90, 90-80, 80-70, 70-60, and 60-50%) of correct classification.

The best separation of healthy controls and CLL using two markers was achieved for combination of CD64 (%) - neutrophil (%) and CD54 (%) - neutrophil (%), thus further confirming observation that lower percentage of neutrophils is associated with higher percentage of CD64 and CD54 markers (Figure 4). Similarly, combination of CD64 (%) - CD54 (%) - neutrophil (%) showed good separation between studied CLL subgroups, achieving a classification error less than 15 % (Figure 5).

### Influence of fMLP and PMA on ROS production by neutrophils

Having identified increased expression of pro-inflammatory surface antigens by neutrophils from the patients, we next sought to investigate the basal and induced ROS generation in neutrophils *ex vivo*. For this purpose, we induced whole blood cells with fMLP (weak chemotactic stimulus) and PMA (non-receptor-dependent activator of protein kinase C) and measured conversion of DHR-123.

**Figure 6:**
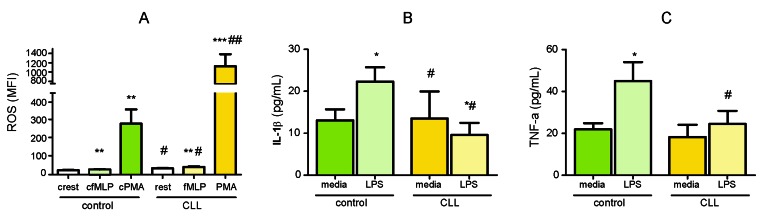
Functional analyses of circulating neutrophils **A.** Basal (rest) and fMLP- and PMA-induced ROS production by neutrophils from the blood of CLL patients (CLL, *n* = 18) and healthy controls (control, *n* = 17). Error bars represent means ± SEM. Induced (*P < 0.05, **P < 0.01, ***P < 0.001) *vs* resting ROS production; (^#^*P* < 0.05, ^##^*P* < 0.01) in CLL *vs* control group. **B.** IL-1β and C.TNF-a release by neutrophils isolated from the blood of CLL patients (CLL, *n* = 16) and healthy controls (control, *n* = 7) after priming with LPS. Error bars are means ± SEM (*P < 0.05, **P < 0.01) *vs* media; (^#^*P* < 0.05, ^##^*P* < 0.01) *vs* control group.

The fraction of neutrophils with spontaneous (resting burst) ROS production was increased in patients with CLL compared to controls (mean MFI; 34.9 *vs* 24.2, *P* < 0.05). The average increase in ROS response to fMLP was detected in CLL group compared to basal secretion level (42.4 *vs* 34.9, *P* < 0.01) and compared to healthy control group (42.4 *vs* 28.7, *P* < 0.05). Despite PMA markedly induced ROS production by neutrophils in both healthy and CLL groups (*P* < 0.01 and *P* < 0.001, respectively), stimulation with PMA has led to a maximum ROS-release in neutrophils from CLL patients that was 4-fold greater than those in healthy controls (1125 *vs* 281, *P* < 0.01) (Figure 6A).

### Surface marker expression on neutrophils after LPS priming

To determine whether the observed phenotypical changes of neutrophils in CLL are associated with altered functional responses of the cells, we primed circulating neutrophils with LPS and measured expression levels of CD54, CD11b, CD62L, CD64, TLR2 and TLR4 using flow cytometry. Phenotypic changes induced by LPS *ex vivo* were selective. LPS upregulated expression of CD11b and TLR2 in healthy cells, and CD11b in CLL group (*P* < 0.05). In opposite, TLR2 expression after LPS exposure was downregulated in neutrophils from CLL patients (Supplementary Figure 6 in on-line Supplement). The expression of other markers was not influenced by LPS exposure (*P* > 0.05).

### IL-1β and TNF-a production in cell culture supernatants after LPS priming and TNF-a levels in serum

We further studied the impact of bacterial LPS on IL-1β and TNF-a release by cultured neutrophils from patients with CLL and healthy subjects. Additionally, we investigated the protein levels of TNF-a in sera of CLL patients and healthy controls.

LPS stimulation of isolated neutrophils from control group induced production of both pro-inflammatory cytokines (*P* < 0.05). In contrast, neutrophils from CLL patients failed to induce either IL-1β or TNF-a release from LPS-stimulated cells. Moreover, LPS exposure decreased production of IL-1β by neutrophils isolated from CLL patients (*P* < 0.05). Both LPS-induced production of IL-1β and TNF-a in CLL group was lower than those released by neutrophils from healthy group (*P* < 0.05) (Figure 6B, 6C).

When comparing the serum levels of TNF-a, higher serum level was observed in CLL patients than in healthy controls (*P* < 0.001) (Supplementary Figure 7 in on-line Supplement).

## DISCUSSION

A growing body of evidences emphasizes the importance of the complex relationship between the CLL clone(s) and its immune environment. The contribution of neutrophils, effectors in both innate and adaptive immunoregulatory networks, to the pathogenesis of CLL was not established yet. To gain more insights on the phenotype of circulating neutrophils in CLL, we studied expression of membrane-bound markers associated with cell activation (CD54, CD11b, CD62L, CD64). Our data demonstrates for the first time that patients with CLL have increased percentages of circulating CD54+ and CD64+ neutrophils and increased CD54 expression. It is tempting to speculate that activated phenotype of neutrophils in CLL, irrespective of infection, is a result of the presence of systemic inflammatory cytokine milieu [13, 14] which largely promotes pro-survival and proliferation signaling in tumor cells [15]. Our data on increased serum concentrations of TNF-a provide further support for the concept of a systemic inflammation in CLL.

Importantly, we detected an increase in the ROS-generating activity in neutrophils from CLL patients suggesting their functionality. CLL patient neutrophils produced markedly more ROS in both resting condition as well as after stimulation with fMLP and PMA *ex vivo* than healthy neutrophils. The increased oxidative potential of these cells could be explained by their altered energy metabolism or chronic activation of the immune system, thus deserving further investigations. Generally circulating neutrophils are a major source of ROS in human blood, having main involvement in microbial defense. While keeping the same absolute number of circulating neutrophils in CLL as in healthy controls, the contribution of increased ROS-generating activity in neutrophils from CLL patients may have significant effects on the pathophysiology of CLL far beyond these cells. In line with our observation, there is already evidence about a robust extracellular release of ROS from neutrophils in CLL exposed to anti-CD20 treatment or αCD20-opsonized CLL cells [16]. The same authors proved that the enhanced ROS production by neutrophils and monocytes in CLL may limit the NK cell-mediated ADCC against CLL cells during anti-CD20 treatment, as NK cell ADCC could be partially restored by anti-oxidative agents [17]. Enhanced ROS production from neutrophils may also participate in tumorigenesis [18], induce mutations and genotoxicity generally [19], contribute to drug resistance and aggressive disease course [20] as well as to a systemic T- and NK-cell dysfunction [21]. More studies are warranted to investigate whether ROS released from activated neutrophils may prevent effective elimination of CLL cells during treatment and contribute to the disease progression as well as perspectives of anti-oxidative drugs to restore the effector function of NK cells in treated patients.

Next, we were interested whether immunophenotype of neutrophils in CLL changes during the disease course. Indeed, significant differences in phenotypic characteristics of neutrophils have been found in our patients with active disease, treatment histories as well as unmutated *IGHV* gene status, mainly by elevation of CD64 and CD54 expression. Of note, upregulation of CD64 and CD54 was associated with low absolute number and low percentage of circulating neutrophils as well as high CLL cells/neutrophils ratio, but not linked to ongoing infection. CD64 (Fcγ receptor I) is expressed by neutrophils when they are activated [22] and serves therefore as a marker for the systemic inflammation and infections [23]. In addition to promoting phagocytosis, CD64 mediates cytotoxicity and activates oxidative burst [24]. CD64 is upregulated in many inflammatory diseases as well as solid tumor cell lines [25]. It has been shown that CD64 expression is also induced by IFNγ [26], a cytokine relevant for development and/or progression of CLL [27]. We therefore suggest that the pro-inflammatory cytokine environment in CLL certainly contributes to the elevation of CD64. So far, several lines of evidence indicate that elimination of CD64-positive macrophages and neutrophils resolves chronic inflammation thus suggesting that this marker may regulate chronicity of inflammation [28, 29]. Taken together, the results of the present study suggest that immunophenotype of neutrophils and particularly CD64 expression is affected by more aggressive disease course. The harm effect of CD64 in CLL, and its relevance to the altered function of circulating neutrophils bearing this receptor remain elusive.

Modulation of neutrophil activation state is accomplished by a number of mechanisms, including neutrophil priming. In this study, the response to bacterial endotoxin was markedly attenuated in the patients’ neutrophils. LPS exposure of neutrophils in CLL has led to the decreased release of two main pro-inflammatory cytokines, IL-1β and TNF-a, comparing to neutrophils from healthy controls. Flow cytometry revealed that LPS priming caused selective downregulation of TLR2 on the surface of neutrophils from CLL patients without influencing the expression of other studied molecules. Thus, neutrophils in CLL failed to mount a standard inflammatory response and exhibited unusual activated immunophenotype with characteristics of suppressed functionality. Such abnormal state of neutrophils might be defined as a tolerance of neutrophils to endotoxin which represents adaptive response of organism that protects against exaggerated inflammation. Persistent inflammation leads to dysfunction of innate immune responses and as a result increased susceptibility to infections. Failure to raise a proper inflammatory reaction might be vital for the antimicrobial defense in patients with CLL and compromise the ability to kill microbial invaders. This is in line with an emerging concept that monocytes in CLL exert primary features of endotoxin tolerance [30, 31]. Similar refractory behaviour, known as ‘tumor tolerance’, has been described in tumor-associated macrophages after repeated contacts with cancer cells [32, 33]. Whether observed functionality on neutrophils is a result of persistent presence of neoplastic cells or ‘real’ endotoxin tolerance is unknown. Further studies are needed to clarify this issue.

In conclusion, our data provide first evidence that neutrophils in CLL are permanently primed and have functional defects. Their phenotype reflects clinical course of the disease and may account for higher susceptibility to bacterial infection in active and treated CLL. The precise mechanisms by which neutrophils contribute to CLL pathogenesis remain unclear and further investigations of neutrophil functions in the context of leukemia and leukemia-related inflammation are needed.

## MATERIALS AND METHODS

### Study population and materials

We analyzed peripheral blood samples from 54 patients with CLL, all diagnosed according to the IWCLL guidelines [34]. Subgroups were formed based on disease activity (sign of progressive/symptomatic disease as defined in the IWCLL guidelines) [34], treatment history and *IGHV* mutational status. Ongoing infections were clinically and laboratory (CRP serum levels, procalcitonin levels, etc.) documented. All previously treated CLL patients were not receiving any treatment during the previous 17 (median, range 1-63) months before sampling; none of the patients received therapy at the time of the blood analysis. For more details about the used treatment in enrolled CLL patients and time between last treatment and the sampling see Supplementary Table 2 in on-line supplement. The clinical characteristic of CLL patients is shown in Table 2.

**Table 2:** Patient characteristics.

*Parameter*	*CLL (n=54)*
Age, years, median (min-max)	68 (50-86)
Gender, n, male/female	29/25
Binet stage (A/B/C/unknown)	23/15/11/5
Disease activity at the time of sampling (yes/no/unknown)	24/29/1
Ongoing infection at the time of sampling (yes/no)	8/46
Treatment (yes/no)*	28/26
*IGHV* gene mutation status (mutated/unmutated/unknown)	22/26/6
Circulating neutrophils Percentage, median (min-max) Absolute number (x10^9^/L), median (min-max)	13.1 (0.05-55.8) 3.7 (0.05-9.8)
Circulating lymphocytes Percentage, median (min-max) Absolute number (x10^9^/L), median (min-max)	78.8 (33.0-97.5) 24.8 (1.2-204.1)
CLL cells Percentage, median (min-max) Absolute number (x10^9^/L), median (min-max)	80.0 (1.3-98.0) 17.6 (0.02-187.1)

*For more information regarding the treatment in previously treated CLL patients see Supplementary Table 2 in on-line Supplement.

Peripheral blood samples from 37 healthy age-matched control subjects (median age 68 yrs; range 38-90 yrs) were taken from members of medical staff or their relatives; all completed a questionnaire regarding their health status. Peripheral blood samples from patients and controls were processed within an hour after collection. Serum samples were aliquoted and stored at -80°C until analysis. All patients and controls provided written informed consent about the usage of peripheral blood for the purpose of this study. The study was approved by the ethics committee of University Hospital and Palacky University Olomouc.

### Neutrophil surface receptor expression measured by flow cytometry

Neutrophils in whole blood were stained with antibodies to the various surface molecules by a direct immunofluorescence technique. To avoid the aberrant sedimentation of granulocytes evident in cancer patients causing the loss of neutrophils during isolation procedure [21], we analyzed neutrophils in whole blood. Viability of neutrophils, assessed by trypan blue exclusion and flow cytometry of propidium iodide-stained cells, was greater than 98%. Briefly, optimal concentrations of monoclonal antibody combinations directed against the following human surface antigens: anti-CD54-FITC (clone HA58), anti-CD11b-PerCP-Cy5.5 (clone ICRF44), anti-CD15-Pe-Cy7 (clone W6D3), anti-CD62L-APC (clone DREG-56), anti-CD64-APC-Cy7 (clone 10.1) (all BioLegend) and anti-CD16-PE (clone B-E16, IQ Products) were incubated with 50 μL of whole blood for 20 minutes at room temperature in the dark. This was followed by lysis of the red blood cells with 2 mL of FACS lysing solution (diluted 1:10 with distilled water; Becton Dickinson) and washing with PBS containing 1% bovine serum albumin (BSA). Neutrophils were identified by their light scatter characteristics and bright immunofluorescence with anti-CD15 and anti-CD16 mAb (Supplementary Figure S1 in on-line supplement). Isotype matched FITC, PE, PerCP-Cy5.5, Pe-Cy7, APC and APC-Cy-7-conjugated irrelevant antibodies (all clones MOPC-21, BioLegend) were used as negative controls. Analysis was performed on a BD FACSCanto II (Becton Dickinson). Data acquisition was performed using BD FACSDiva software (Becton Dickinson). Flow cytometry data were analyzed using the FlowJo vX0.7 software (Tree Star, Inc, San Carlos, CA). In all experiments, a minimum of 10,000 events was counted. Results are expressed as the percentage and MFI of the cells for each examined marker.

### Oxidative burst assay

Neutrophil oxidative stress capacity was analyzed using dihydrorhodamine 123 (DHR-123) (Sigma-Aldrich), a nonfluorescent compound which accumulates in mitochondria and is converted to the highly fluorescent rhodamine-123 by the action of oxidative stress. Briefly, the tubes with whole blood were prelabeled with CD15 and incubated with 20 μM DHR-123 (stock solution 5.7 mM DHR-123 in dimethyl sulphoxide, DMSO) in the dark at 37°C for 20 minutes. Then either 400 nM N-Formyl-Met-Leu-Phe (fMLP, Sigma-Aldrich, 11.4 mM stock solution in DMSO) or 2 μM Phorbol 12-myristate 13-acetate (PMA, Sigma-Aldrich, 1.6 mM stock solution in DMSO) was added and the cell suspensions were incubated in the dark at 37°C for further 20 minutes. For the negative control, whole blood cells were incubated with DMSO. The red blood cells were lysed with FACS lysing solution.

### Isolation of peripheral blood neutrophils and priming with LPS

Peripheral blood samples were collected in tubes containing EDTA and processed within an hour after collection. Neutrophils were isolated by density centrifugation using the Histopaque gradient technique (Sigma-Aldrich) according to the manufacturer’s protocol. Contaminating erythrocytes were removed with FACS lysing solution and washed with PBS containing 1% BSA. Viability of neutrophils after purification, assessed by trypan blue exclusion and flow cytometry of propidium iodide-stained cells, was greater than 96%.

For priming of isolated neutrophils, cells (1.5 × 10^6^ cells/mL) were cultured at 37°C in RPMI-1640 (Sigma-Aldrich) supplemented with 10% heat-inactivated fetal calf serum, 2 mM L-glutamine, 1 mM sodium pyruvate, 5 mM HEPES, 100 U/mL of penicillin, 100 µg/mL of streptomycin in the absence or presence of 100 ng/mL LPS (*Escherichia coli* 0111:B4, Sigma-Aldrich) in a total volume of 500 µL for 4 hours. After stimulation, neutrophils were harvested, washed once with cold PBS and stained with anti-CD54, CD64, CD11b, CD15, TLR2 (clone TL2.1), TLR4 (clone HTA125) (all BioLegend) and analyzed with flow cytometer similarly to the analysis of surface antigens. Supernatants were stored at -80°C until analysis of cytokine production.

### ELISA in supernatants and serum

Enzyme-linked immunosorbent assay (ELISA) kits were used to measure the levels of IL-1β and TNF-a (BioLegend) in cell-culture supernatants; Human TNF-a Quantikine HS ELISA (R&D Systems, Inc.) for the levels of TNF-a in serum. The assays were performed following the manufacturer’s recommendations and absolute cytokine levels were calculated based on comparison to assay performance in the presence of known quantities of recombinant cytokine standards. The detection limits of ELISA were 0.5 and 2 pg/mL for IL-1β and TNF-a (BioLegend), and 0.19 pg/mL for TNF-a (R&D Systems, Inc), respectively.

### Statistical analyses

Statistical analyses were performed using GraphPad Prism 5·0 (GraphPad Software, La Jolla, CA, USA) and R statistical software package (http://www.r-project.org/). Subsequently, the Mann-Whitney U-test was used to compare the expression of markers between groups. Differences between unstimulated and stimulated samples from the same subject were calculated using the Wilcoxon non-parametric test for paired samples. *P* values < 0.05 were considered significant. Multilinear Discriminant Analysis (MDA) and extended Support Vector Machine (kSVM) [35] were used to calculate the class probabilities for combinations of two/three markers using R statistical software with package Caret (http://topepo.github.io/caret/index.html).

## SUPPLEMENTARY MATERIALS FIGURES AND TABLES



## Abbreviations

CLL: chronic lymphocytic leukemia
ROS: reactive oxygen species
ADCC: antibody-dependent cell-mediated cytotoxicity
MFI: median fluorescence intensity
FcR: Fc receptor
fMLP: N-Formyl-Met-Leu-Phe
PMA: Phorbol 12-myristate 13-acetate
DHR-123: Dihydrorhodamine 123
LPS: Lipopolysaccharide
IWCLL: International Workshop on Chronic Lymphocytic Leukemia
PBS: Phosphate-buffered saline
BSA: bovine serum albumin
ANC: Absolute neutrophil count
