# Supplementary material for: Neutrophils in chronic lymphocytic leukemia are permanently activated and have functional defects
Source: Oncotarget. 2017 Aug 8;8(49):84889–901. doi: 10.18632/oncotarget.20031 (PMC5689581; doi:10.18632/oncotarget.20031)
Supplement: Supplementary file 1 [file oncotarget-08-84889-s001.pdf]

Neutrophils in chronic lymphocytic leukemia are permanently activated and have functional defects

SUPPLEMENTARY MATERIALS

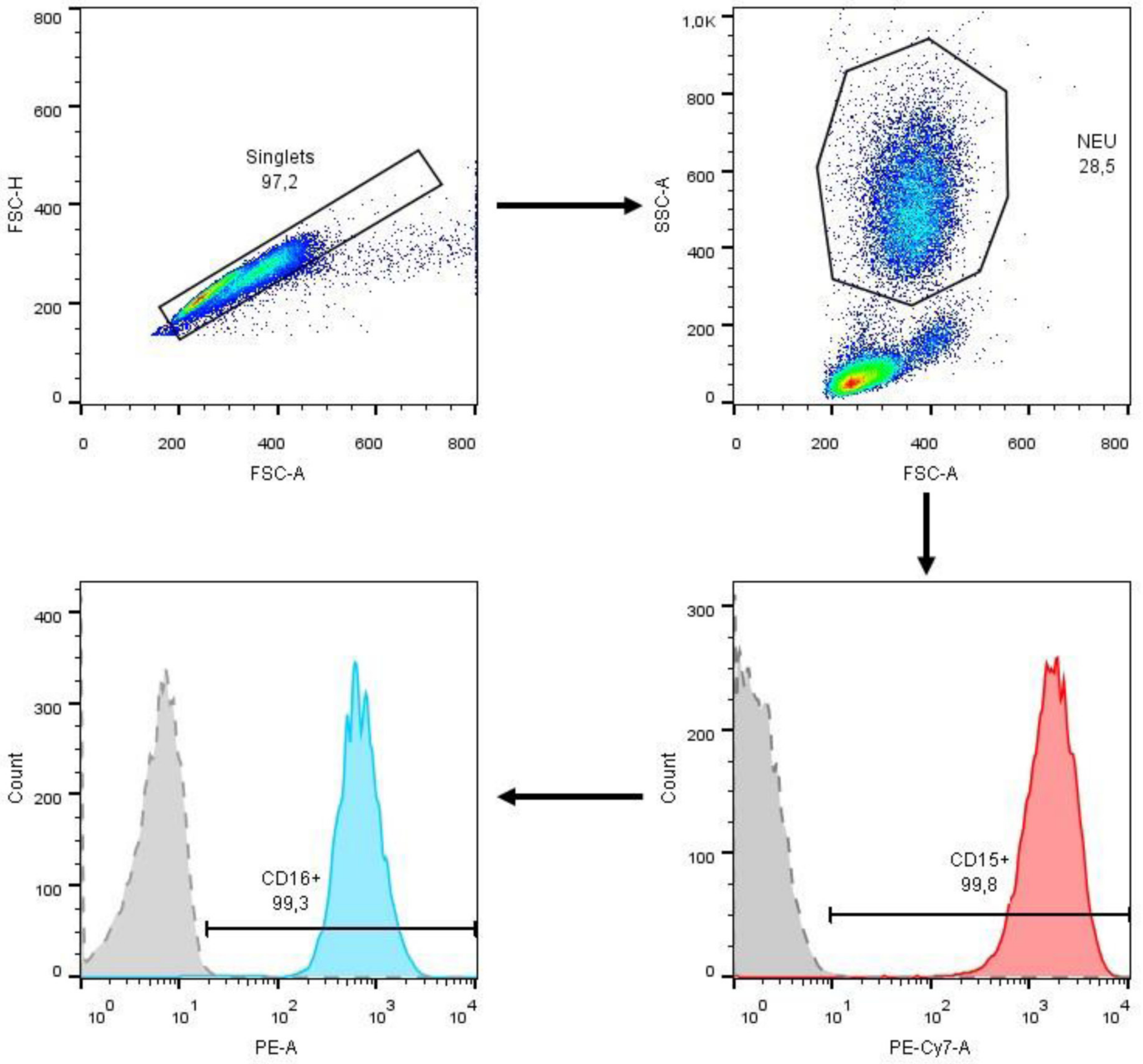

Supplementary Figure 1: Gating strategy for neutrophils. Cell doublets were excluded by displaying the forward scatter (FSC) height values vs FSC area values. Population of neutrophils was identified based on FSC and side scatter (SSC) properties and bright fluorescence signal of conjugated anti-CD15 and anti-CD16 antibodies. Isotype matched irrelevant antibodies were for elimination of negative signals (displayed with grey peaks). Numbers in figures indicate the percentage of gated cell populations (% of singlets, % of neutrophils and % of CD15+ CD16+ neutrophils, respectively).

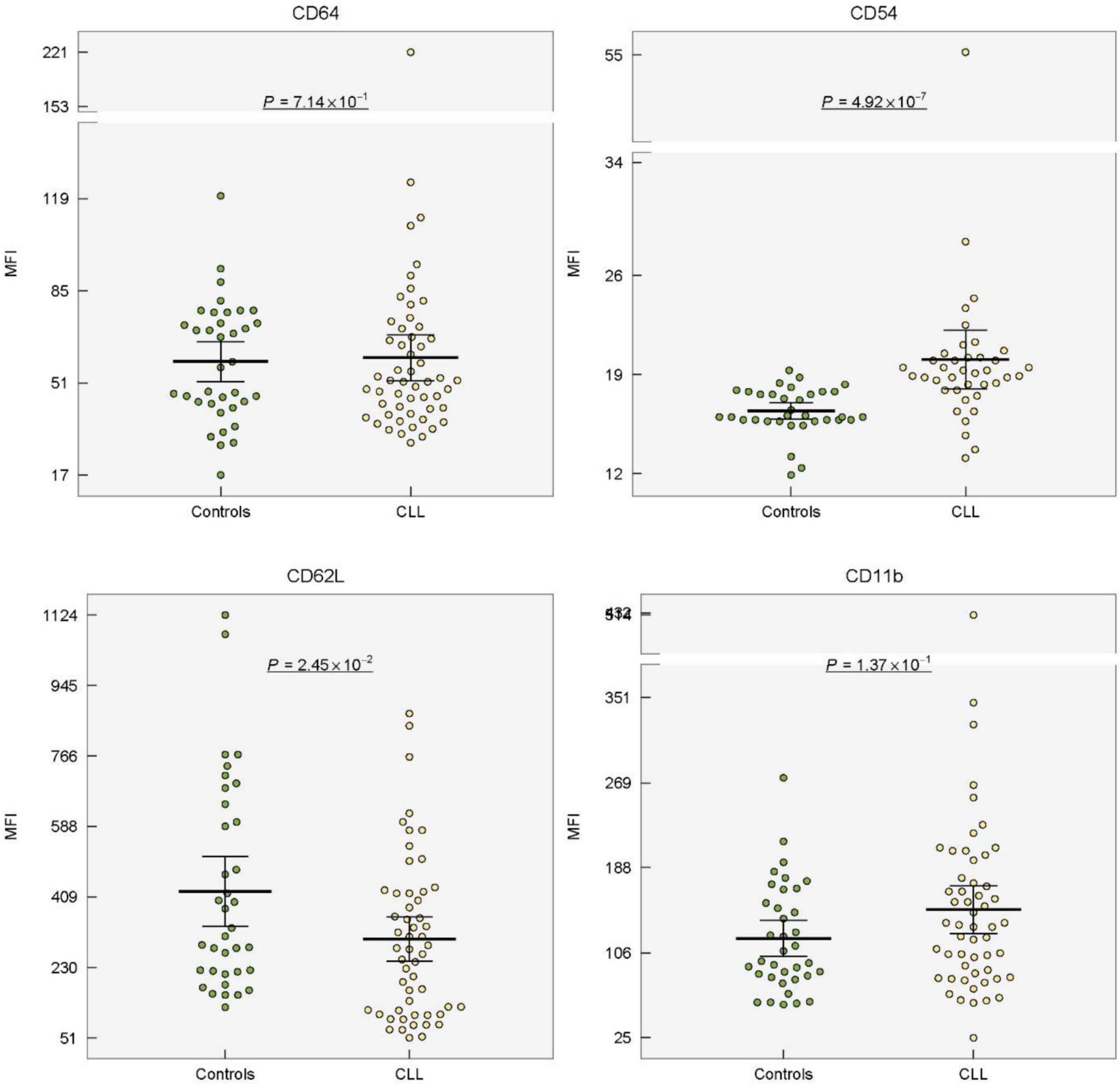

Supplementary Figure 2: Distribution of median expression intensities (MFI) of surface markers (CD64, CD54, CD62L, CD11b) on circulating neutrophils from patients with CLL and healthy controls. Group means are indicated by horizontal bars, error bars indicate 95%CI; *P* values for differences between two groups are stated.

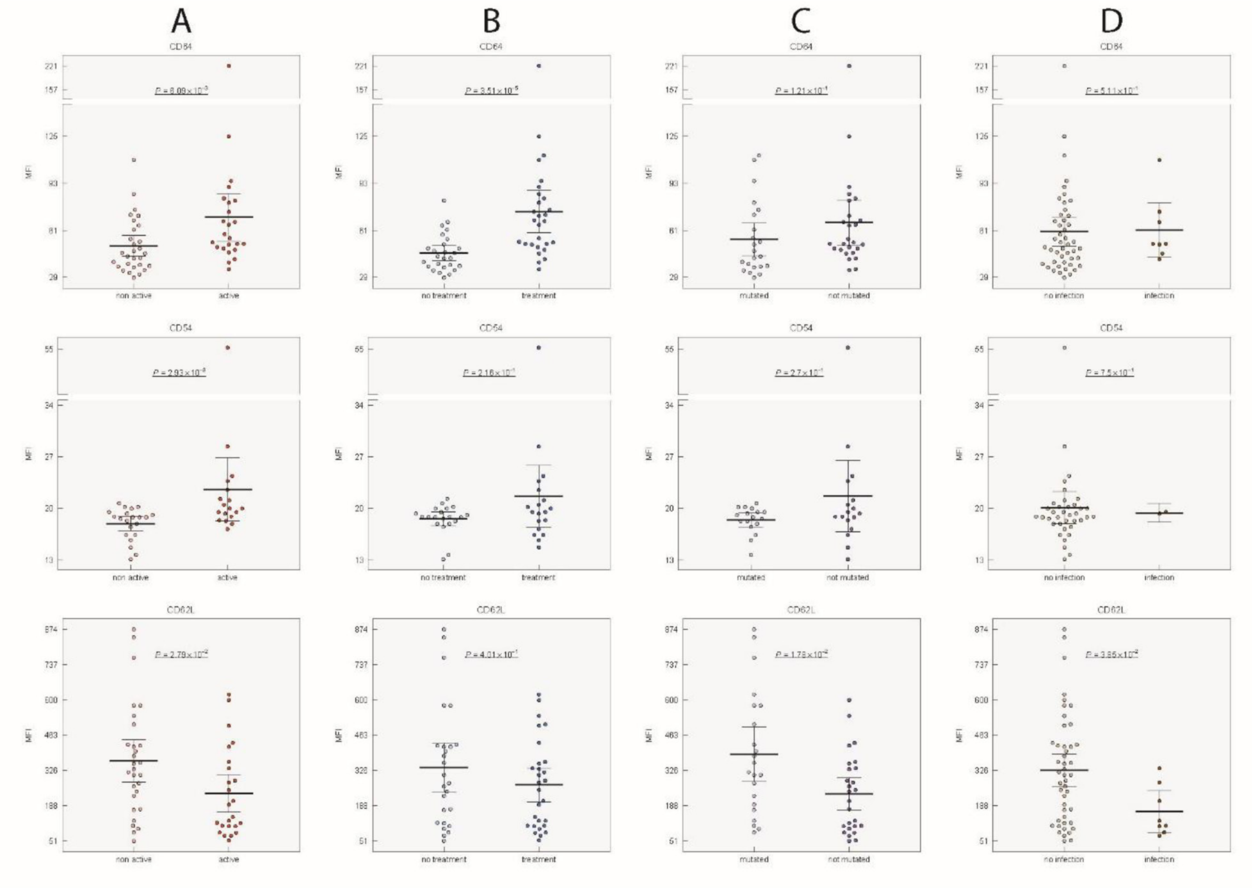

Supplementary Figure 3: Distribution of surface markers (CD64, CD54, CD62L) median expression intensities (MFI) on neutrophils in CLL subgroups: A) non-active vs active disease, **B**) untreated vs treated disease, **C**) mutated vs unmutated *IGHV* gene status, **D**) CLL patients without infection vs with ongoing infection**.** Group means are indicated by horizontal bars, error bars indicate 95%CI; *P* values for differences between two groups are stated.

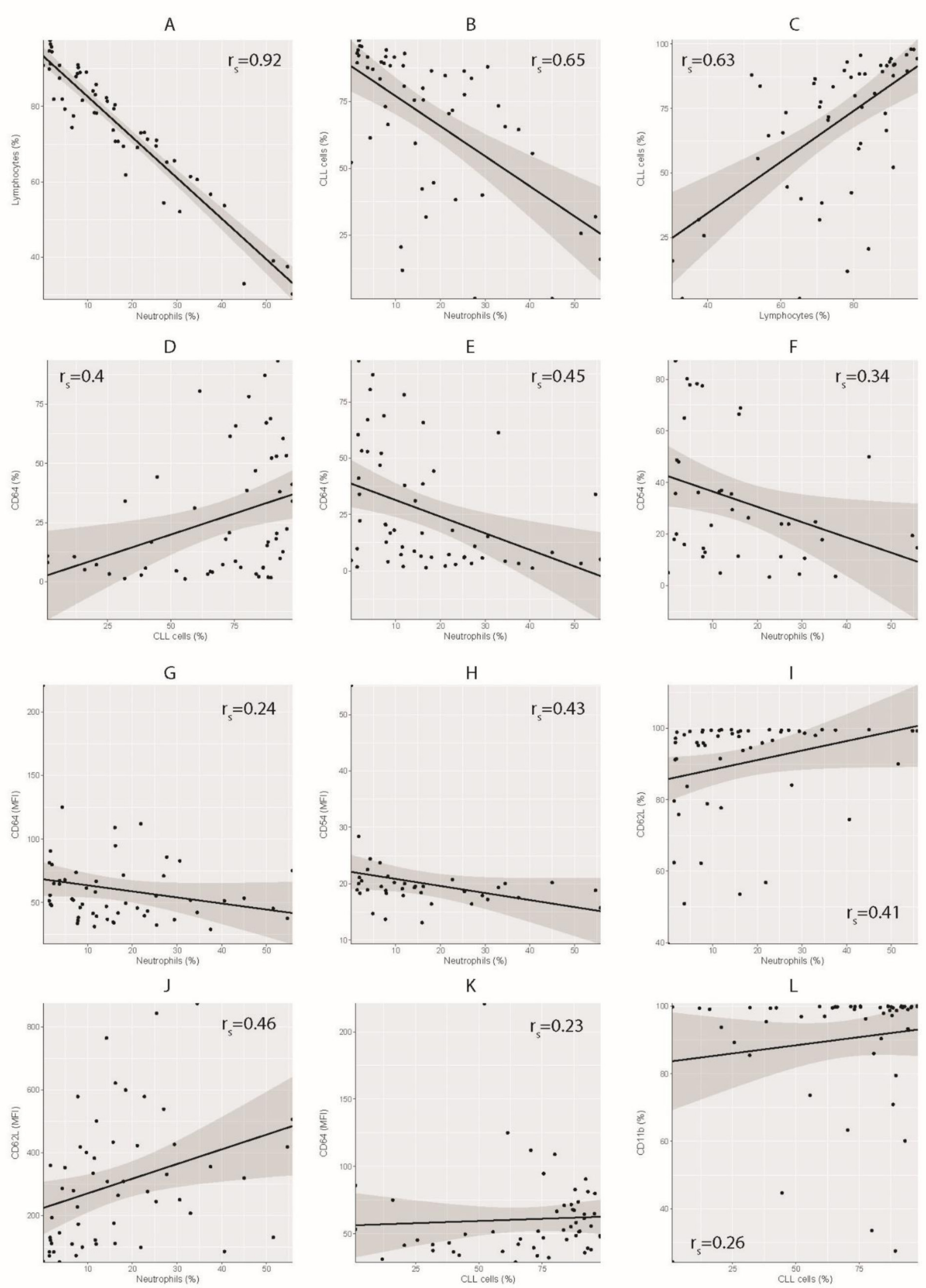

Supplementary Figure 4: Association between surface markers and percentage of circulating neutrophils, lymphocytes and CLL cells in patients with CLL: A) lymphocytes (%) vs neutrophils (%), **B**) CLL cells (%) vs neutrophils (%), **C**) CLL cells (%) vs lymphocytes (%), **D**) CD64 (%) vs CLL cells (%), **E**) CD64 (%) vs neutrophils (%), **F**) CD54 (%) vs neutrophils (%), **G**) CD64 (MFI) vs neutrophils (%), **H**) CD54 (MFI) vs neutrophils (%), **I**) CD62L (%) vs neutrophils (%), **J**) CD62L (MFI) vs neutrophils (%), **K**) CD64 (MFI) vs CLL cells (%), **L**) CD11b (%) vs CLL cells (%).

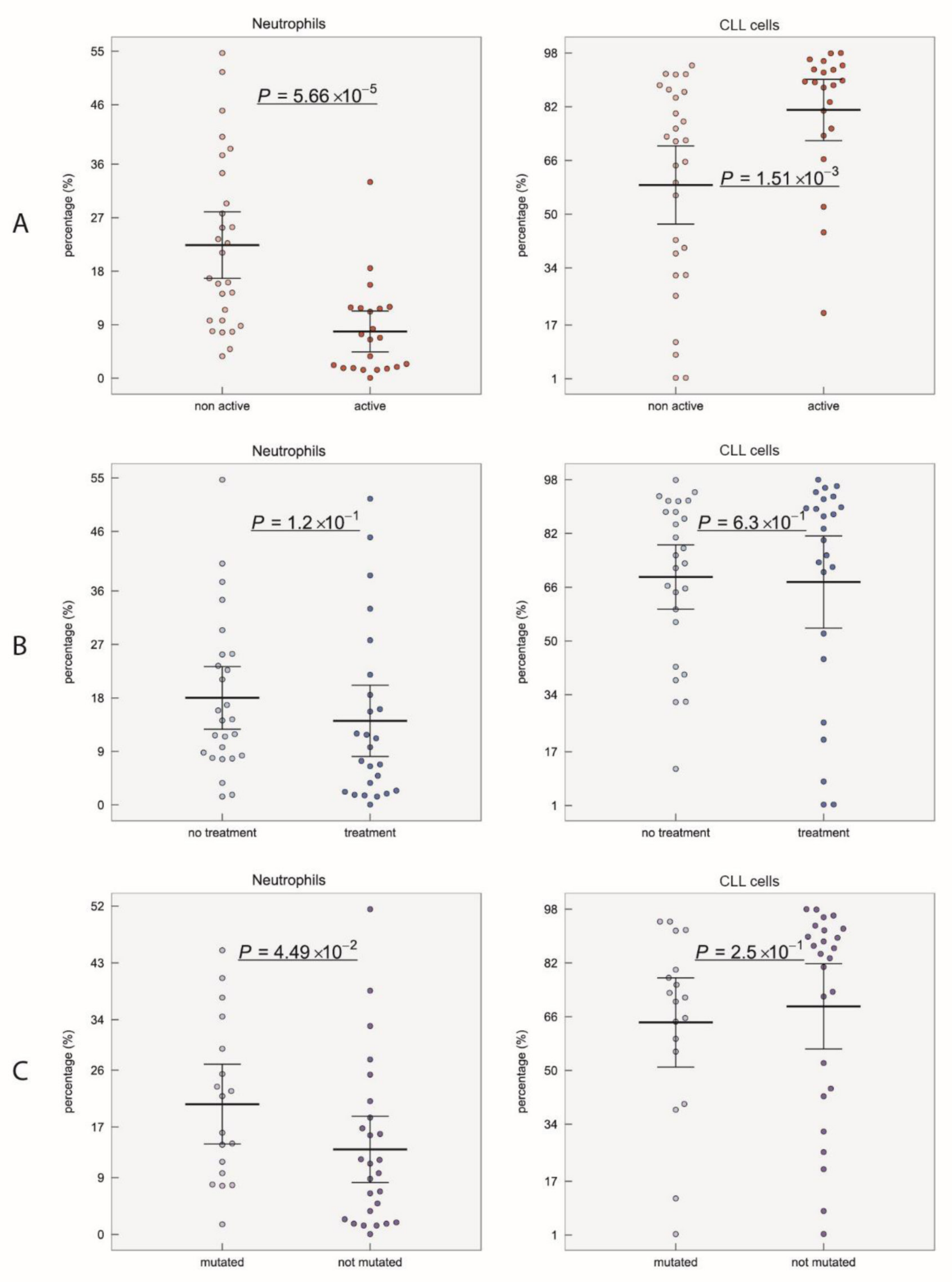

Supplementary Figure 5: Distribution of percentages of circulating neutrophils and CLL cells in **A**) non-active vs active disease, **B**) untreated vs treated disease, **C**) mutated vs unmutated *IGHV* gene status**.** Group means are indicated by horizontal bars, error bars indicate 95%CI; *P* values for differences between two groups are stated.

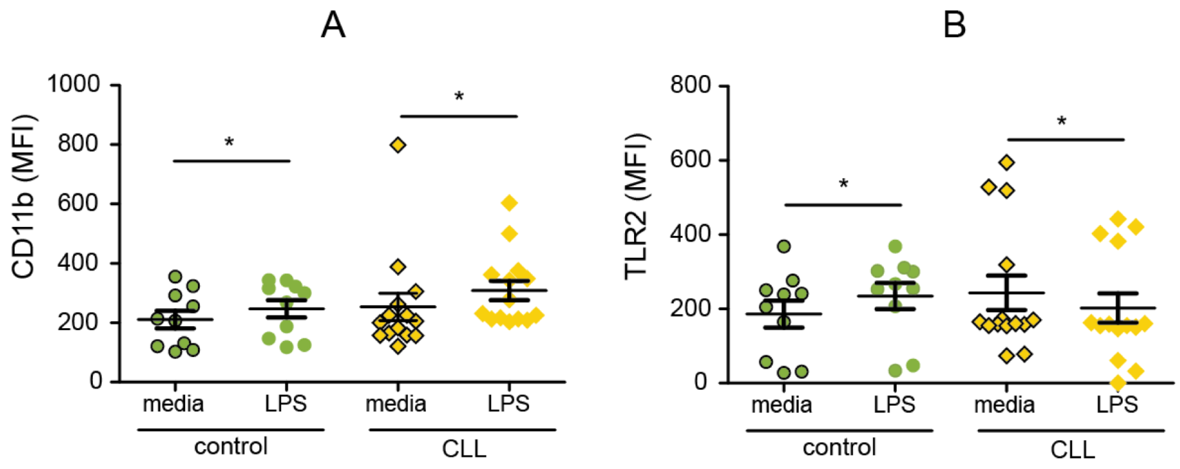

Supplementary Figure 6: Influence of LPS exposure on CD11b and TLR2 expression on neutrophils from CLL. The cell surface expression of CD11b and TLR2 was measured on neutrophils from CLL patients (CLL, n=14) and healthy controls (control, n=10) after LPS exposure. The data are presented as median fluorescence intensity (MFI) and reported as means ± SEM (*P<0.05).

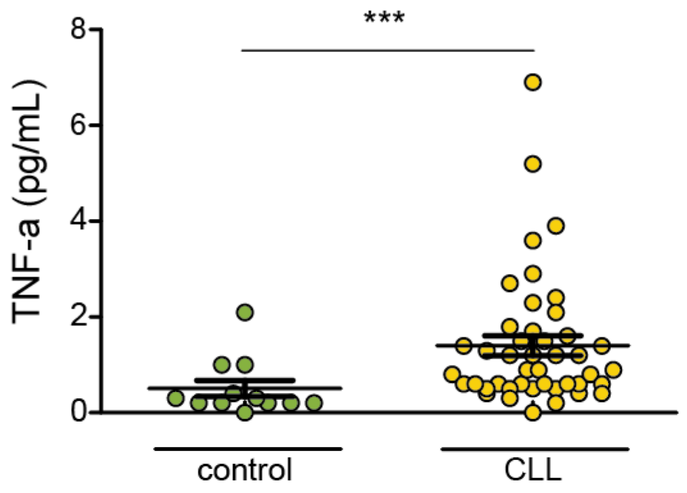

Supplementary Figure 7: Serum TNF-a levels in CLL patients. The TNF-a levels were investigated in serum from CLL patients (CLL, n=44) and healthy control subjects (control, n=12) and reported as means ± SEM (***P<0.001).

Supplementary Table 1: Relative and absolute neutrophil counts and expression levels of surface markers on neutrophils in: A) Healthy controls *vs* untreated CLL, B) Healthy controls *vs* treated CLL

Marker	*Mean (95% CI)*		*FC*[#]	*P*
**A**	***Healthy controls***	***CLL untreated***		
CD64 (%)	6.89 (3.73-10.0)	16.5 (9.12-23.9)	2.96	2.43×10^{-3}
CD54 (%)*	16.2 (11.5-20.9)	23.3 (14.0-32.5)	1.43	0.20
CD62L (%)	98.3 (97.3-99.3)	93.0 (88.3-97.6)	0.91	5.92×10^{-3}
CD11b (%)	96.5 (93.8-99.3)	94.6 (90.3-98.9)	0.99	0.48
CD64 (MFI)	58.8 (51.4-66.3)	45.6 (40.5-50.8)	0.74	0.01
CD54 (MFI)*	16.4 (15.9-17.0)	18.6 (17.6-19.5)	1.17	1.68×10^{-5}
CD62L (MFI)	423 (334-512)	338 (243-433)	0.91	0.20
CD11b (MFI)	120 (103-138)	156 (118-193)	1.36	0.08
Neutrophils (%)	58.4 (56.2-60.7)	18.0 (12.7-23.3)	0.25	4.4×10^{-11}
Lymphocytes (%)	28.8 (26.9-30.8)	76.6 (71.0-82.2)	2.78	2.38×10^{-11}
ANC ($x10^9$/L)	4.82 (4.09-5.55)	5.01 (4.14-5.87)	0.86	0.89
CD64+ ANC ($x10^9$/L)	0.40 (0.08-0.71)	0.94 (0.29-1.59)	2.24	0.02
CD54+ ANC ($x10^9$/L)	0.59 (0.33-0.85)	1.09 (0.54-1.64)	2.07	0.06
CD62L+ ANC ($x10^9$/L)	4.79 (4.07-5.51)	4.56 (3.82-5.31)	0.78	0.44
CD11b+ ANC ($x10^9$/L)	4.81 (4.08-5.54)	4.76 (3.90-5.62)	0.79	0.61
**B**	***Healthy controls***	***CLL treated***		
CD64 (%)	6.89 (3.73-10.0)	35.5 (24.1-46.9)	10.42	4.13×10^{-7}
CD54 (%)*	16.2 (11.5-20.9)	42.8 (29.9-55.8)	2.96	2.49×10^{-4}
CD62L (%)	98.3 (97.3-99.3)	87.8 (81.2-94.4)	0.96	4.27×10^{-5}
CD11b (%)	96.5 (93.8-99.3)	86.6 (77.3-95.9)	0.92	0.02
CD64 (MFI)	58.8 (51.4-66.3)	73.8 (59.5-88.1)	1.17	0.08
CD54 (MFI)*	16.4 (15.9-17.0)	21.7 (17.4-25.9)	1.21	7.08×10^{-5}
CD62L (MFI)	423 (334-512)	269 (203-336)	0.83	0.01
CD11b (MFI)	120 (103-138)	140 (111-170)	1.11	0.45
Neutrophils (%)	58.4 (56.2-60.7)	15.9 (9.81-22.0)	0.20	3.66×10^{-11}
Lymphocytes (%)	28.8 (26.9-30.8)	73.8 (66.6-81.0)	2.69	6.23×10^{-11}
ANC ($x10^9$/L)	4.82 (4.09-5.55)	3.42 (2.76-4.09)	0.60	5.94×10^{-3}
CD64+ ANC ($x10^9$/L)	0.40 (0.08-0.71)	1.18 (0.73-1.63)	5.27	5.94×10^{-3}
CD54+ ANC ($x10^9$/L)	0.59 (0.33-0.85)	1.41 (0.94-1.89)	3.77	1.93×10^{-3}
CD62L+ ANC ($x10^9$/L)	4.79 (4.07-5.51)	3.13 (2.44-3.82)	0.51	1.56×10^{-3}
CD11b+ ANC ($x10^9$/L)	4.81 (4.08-5.54)	3.05 (2.31-3.78)	0.50	2.04×10^{-3}

Legend: ANC: Absolute neutrophil count. The normal range for the ANC = 1.5 to $8.0x10^9$/L.
[#]FC (Fold Change) between group medians
*CD54 available in 39 patients and 34 controls

Supplementary Table 2: Type and line of treatment in CLL patients with treatment history

Patient	Type and line of treatment	last treatment before sampling
P1	RCD/FCR (1st line)	24 months
P2	FCR (1st line), FCR (2nd line)	36 months
P3	RCD (1st line)	24 months
P4	FCR (1st line)	63 months
P5	RCD (1st line)	4 months
P6	FCR (1st line), FCR (2nd line), R-bendamustine (3rd line)	27 months
P7	FCR (1st line)	12 months
P8	FCR (1st line), CHOP (2nd line), CFA + ETO + dexamethasone (3rd line)	60 months
P9	FCR (1st line)	20 months
P10	FCR (1st line)	53 months
P11	FCR, R-dexamethasone, CFA (1st line)	38 months
P12	FCR Q-lite (1st line), alemtuzumab (2nd line)	7 months
P13	COP (1st line), FCR (2nd line), RCD (3th line), R-bendamustine (4th line), alemtuzumab (5th line), RCD (6th line)	2 months
P14	FCR (1st line)	25 months
P15	RCD (1st line)	8 months
P16	FCR (1st line)	17 months
P17	R-CHOP (1st line), FCR (2nd line), RCD + R-bendamustine (3th line)	1 month
P18	R-bendamustine (1st line), FCR Q-lite (2nd/3th lines), FCR (3th line)	7 months
P19	FCR (1st line), R-bendamustine (2nd line)	7 months
P20	chlorambucil, CFA + prednisone (1st line)	1 month
P21	R-bendamustine (1st line), FCR Q-lite (2nd line)	7 months
P22	Ofatumumab + bendamustine (1st line)	28 months
P23	FCR Q-lite (1st line), FCR Q-lite (2nd line), RCD (3th line)	12 months
P24	FCR Q-lite (1st line)	46 months
P25	FC (1st line), RCD (2nd line), R-bendamustine (3th line)	12 months
P26	R-bendamustine (1st line)	6 months
P27	FCR (1st line), FCR (2nd line)	16 months
P28	FCR (1st line), FCR Q-lite (2nd line)	40 months

Legend: fludarabin (F), cyclophosphamide (C), rituximab (R), dexamethasone (D), high-dose cytarabine and mitoxantrone (HAM)
